# Integrated cognitive behavioral intervention for functional tics (I-CBiT): case reports and treatment formulation

**DOI:** 10.3389/fped.2023.1265123

**Published:** 2023-11-16

**Authors:** Amanda Maxwell, Jade-Jocelyne Zouki, Valsamma Eapen

**Affiliations:** ^1^ Discipline of Psychiatry and Mental Health, UNSW School of Clinical Medicine, University of New South Wales, Kensington, NSW, Australia; ^2^ Centre for Social and Early Emotional Development and School of Psychology, Deakin University, Geelong, VIC, Australia

**Keywords:** integrated intervention, functional tic like behavior, Tourette syndrome, functional movement disorders, premonitory urge, autism spectrum disorder (ASD)

## Abstract

**Introduction:**

The onset of the COVID-19 pandemic saw a global surge in functional tic-like behaviors (FTLBs). FTLBs are unique from primary tic disorders. They are thought to manifest through a complex interplay between environmental and personal factors, including the stress-arousal system, and are characterized by their sudden and explosive onset. Accordingly, common interventions for tic disorders show limited efficacy in this population. We present an Integrated Cognitive Behavioral Intervention for Functional Tics (I-CBiT) that uses an urge acceptance model to manage tics and related stress and anxiety.

**Methods:**

We describe the treatment outcomes of eight young people presenting with new and sudden onset FTLBs who underwent I-CBiT, which integrates traditional behavioral tic interventions with third-wave cognitive behavioral therapies. All cases completed the three-phase intervention involving core components of psychoeducation, exposure and response prevention with urge acceptance, sensory grounding strategies, and cognitive behavioral intervention targeting the stress-arousal system. Tic severity and impairment were assessed prior to treatment and at completion.

**Results:**

All cases showed a significant reduction in tic severity post I-CBiT and an improvement in overall daily living function. These cases highlight the role of urge acceptance in managing both tic urges and the underlying stress-arousal system to bring about long-term change.

**Conclusion:**

We demonstrated the efficacy of I-CBiT for managing FTLBs. Our findings illustrate the importance of treating underlying stress and anxiety in this population and, therefore, a need for greater interaction between multidisciplinary services in managing FTLBs to comprehensively cover the varied symptom presentations linked to thoughts, emotions, bodily sensations, and stress responses.

## 1. Introduction

Since the onset of the COVID-19 pandemic, there has been a surge in the presentation of Functional (Neurological) Movement Disorder (FND) globally (1, 2), with one clinic in the United States reporting a rise in FND diagnoses by ∼60% in 2020 compared to the previous year (1). This increase is even higher (∼90%) in the pediatric population (1), with a spike in the number of patients presenting with sudden onset functional tic-like behaviors (FTLBs), appearing to coincide with the pandemic (3, 4). Indeed, a specialist tic clinic in Australia reported a significant rise in FTLBs during 2020 and 2021 by 10.6% and 36%, respectively, compared to 2% in 2018 (3).

Although FTLBs are often difficult to discriminate from primary tics seen in persistent tic disorders such as Tourette syndrome, there are some stark epidemiological and phenomenological differences (3, 5), such as more females, later age at tic onset (>12 years), lack of typical waxing-and-waning course (5–8), and greater mean tic severity and related pali/echo/copro-like phenomena (5). They often present with a more severe initial presentation, involving hospitalization and emergency department (ED) visits, and greater impairment in school functioning and peer/family relationships (3, 4). Additionally, the premonitory urge preceding FTLBs typically differs, with patients often describing these sensory phenomena as whole-body pressure or a pulse of energy (9) rather than tension isolated to the specific anatomical region where the tic is generated (10, 11). A higher prevalence of autism spectrum disorder (ASD) and psychiatric symptoms, including comorbid anxiety and depression, are also observed (3, 5). In addition, the presentation of preceding stressful life events, self-harm behaviors (e.g., cutting), suicidal ideation, and the presence of other functional or somatic symptoms are considered minor criteria for the diagnosis of FTLBs (12).

With the increase in FTLB presentations, the need for viable interventions for these historically difficult-to-treat symptoms is evident. Preliminary findings show minimal or no clinical improvement with standard pharmacological treatments (3, 4, 9). Further, while the applicability of Comprehensive Behavioral Intervention for Tics [CBIT (13)] for FTLBs is yet to be reported, our clinical experience indicates that it has limited long-term efficacy, and symptom replacement is not uncommon following the improvement of tics. An explanation for this may be that CBIT is largely a behavioral intervention aimed at reducing tics without specifically targeting the preceding premonitory urge or any comorbid psychopathology underlying the urge and related tic expression (14). Further, while FTLBs share many common features with primary tic disorders, they are suggested to have unique psychological and pathophysiological underpinnings (15) and are best conceptualized using a biopsychosocial model (16, 17). In particular, the stress-arousal system likely plays an important role both in precipitating and maintaining FTLBs (18, 19).

The role of stress and anxiety in precipitating and maintaining complex tic presentations has been previously reported (12, 20, 21). For example, Robinson and Hedderly (21) highlighted the role of anxiety and panic in “tic attacks” (21). In their case report, the authors demonstrated the utility of managing co-occurring FND symptoms (“tic attacks”) by modifying attention, worry processes, and negative beliefs related to symptom presentation using a novel cognitive behavioral therapy-based approach, integrating meta-cognitive and cognitive-behavioral techniques. More recent reports emphasize the role of anxiety in complex tic presentations, noting that FTLBs often co-exist alongside neurodevelopmental tics and that this dual diagnosis has increased in recent years and/or been previously underestimated (20, 22, 23).

Here, we report the clinical outcomes of eight young people with sudden onset FTLBs who were treated using an Integrated Cognitive Behavioral Intervention for Functional Tics (I-CBiT). This novel intervention draws on traditional behavioral tic interventions yet integrates third-wave cognitive behavioral therapies, highlighting the importance of a person's relationship to their thoughts, emotions, and bodily sensations, including stress responses (24).

## 2. Materials and methods

### 2.1. Inclusion criteria

All cases presented to our clinic between 2019 and 2023 and met criteria for FTLBs based on current guidelines (12). Specifically, all cases demonstrated: 1) rapid onset and evolution of symptoms; 2) at least 4/9 phenomenological criteria; and 3) tic onset at >12 years old (in 6/8 cases). Those not meeting the latter criterion met an aforementioned minor criterion (see 12). Diagnoses were made by a clinical psychologist (AM) with confirmation by a neurologist (cases 1, 2, 4, 6), psychiatrist (cases 2, 3, 5, 8), or pediatrician (case 7). See Table 1 for case-specific information regarding medical/family/psychosocial history, clinical presentation, and treatment course. Consent was obtained according to the Declaration of Helsinki. Informed written consent was obtained for all cases.

**Table 1:** Individual case characteristics and I-CBiT treatment course.

Case	Age at tic onset	Prior tic/Age	Risk factor^a^	Tic manifestation	Tic frequency pre/post-treatment; Suppressibility pre/post-treatment^b^	YGTSS (pre; post-treatment)^c^	Patient-specific modifications	Session number
1	13	No	GAD, social anxiety, alexithymia, injury (ACL tear)	Wink, shoulder shrug, throw objects, punch family/self; gun gesture + vocalization “terrorist”; coprolalia	1 min/1 tic every few months (tic free year); 2 min/∼1 h+	42/50; 8/50	School liaison & family sessions; suicide risk management; psychiatry	P1 = 3 P2 = 9 P3 = 14
2	13	Yes/5	MDD, GAD, social anxiety, perfectionism, panic, functional “numbness”, OCBs, family Hx PTSD	Hit, kick, head-bang, copropraxia; echo/coprolalia	10 s/no tics present; 6 min/∼1 h+	47/50; 0/50	School liaison & parent sessions; HRT during tic suppression	P1 = 3 P2 = 6 P3 = 12
3	14	No	ASD (lvl 1), OCBs Hx (hair pulling), panic, family Hx OCBs	Blink, grind teeth, shoulder shrug, jaw protrusion, hold legs/knees to chest, clap, fall down, hit self, kick, throw objects; squeak, vocalization “are you a bird?”, coprolalia	1–2 min/1 tic per 2–3 months; 1 min/∼1 h+	28/50; 4/50	Psychoeducation; social problem-solving; ASD assessment; parent sessions	P1 = 3 P2 = 7 P3 = 8
4	16	No	GAD, MDD, social anxiety, trauma, dissociation, panic, functional seizures, perfectionism	Head jerk, shoulder shrug, hit self, hit head on wall, stab self with pencil; coprolalia	1 min/1–2 per-day; 58 s/18.46 min	48/50; 14/50	School liaison; psychoeducation & CBT for other FND symptoms (functional seizures/limb weakness/somatic pain); suicide risk management	P1 = 2 P2 = 12 P3 = 16
5	14	Yes/11	GAD, self-harm behaviors, panic, perfectionism, OCBs, Hx selective mutism, family Hx ASD	Wink, nod, facial tics, kick, hit, throw objects, jump, copropraxia; change in tone/accent, coprolalia	30 s/1–2 h; 1.22 min/∼1 h+	26/50; 16/50	Parent sessions; self-esteem; gender & sexuality sessions; goal setting around identity; social media usage	P1 = 2 P2 + P3 = 16
6	14	No	Social anxiety, hyperactivity/inattention (undiagnosed), alexithymia	Nod, wink, throw pens, slap, stomp feet; pop + click sounds, vocalization “woo”, “love you”, “cheese”, coprolalia	5–10 min (school) & 20–30 min (home)/2 h (2–3 tic free days); 5 min/∼1 h+	30/50; 17/50	NA	P1 = 2 P2 + P3 = 9
7	13	No	GAD, performance anxiety, perfectionism, alexithymia	Bend over, nod + tense head, nod head to the left, hit/touch head to wall	4 s/tic-free weeks or 1 tic per-hr; 11 s/∼1 h+	18/50; 4/50	School liaison & family sessions	P1 = 2 P2 + P3 = 12
8	20	No	ASD (lvl 2), MDD, GAD, social anxiety, OCBs, panic, perfectionism, gastro symptoms, endometriosis/pelvic pain	Whole body movements, hit self + others, throw items, sit down when crossing roads, echopraxia echo/coprolalia, throat clearing, snort	5 sec/1 tic per month; 4 s/∼1 h+	41/50; 5/50	Family sessions; GP & psychiatrist liaison; psychoeducation & CBT for somatic gastro symptoms	P1 = 2 P2 = 14 P3 = 10

Age is reported in years.

^a^ Risk factors related to presentation of functional symptoms.

^b^ YGTSS was collected at final I-CBiT session. All cases were seen post I-CBiT treatment. Tic frequency noted in results reflects tic presentation at end of Phase 3 of I-CBiT.

^c^ Total tic severity score as indicated by the YGTSS; GAD, generalized anxiety disorder; ACL, anterior cruciate ligament; MDD, major depressive disorder; OCB, obsessive compulsive behaviors; Hx, history; ASD, autism spectrum disorder; HRT, habit reversal training; YGTSS, yale global tic severity scale; IVIG, intravenous immune globulin; FND, functional neurological disorder; P, phase; ∼1 h+, time at recording stopped. Time of suppression may surpass this time.

### 2.2. Patient characteristics and treatment outcomes

#### 2.2.1. Case 1

A 13-year-old female presented with simple motor tics following a holiday where she sustained an anterior cruciate ligament injury (ACL tear). Within a few months, loud coprolalia, self-injurious behaviors, complex orchestrated motor/vocal tic sequences, and tic attacks emerged. History included generalized anxiety disorder (GAD) and alexithymia. Tic onset was preceded by a move to Australia from abroad, resulting in losing friends and a much-loved pet and difficulties adapting socially to a private girls' school. Tics showed a marked reduction after 12 sessions of I-CBiT over three months, with minimal motor tics during stressful periods (exam week). As tics improved with treatment, she presented with a marked drop in mood, increased anxiety, and suicidal ideation, for which Fluoxetine was commenced. Mood stabilized after a further six months. School functioning improved, and no suicide risk was present.

#### 2.2.2. Case 2

A 13-year-old female presented with a sudden onset of explosive motor tics, for which she attended the local ED. Within days, tics progressed to complex vocalizations followed by self-injurious and dystonic tics, reducing school attendance and social functioning. She presented as a high achiever with perfectionist tendencies in both school and sports, playing at a state level. History included GAD, social anxiety, major depressive disorder (MDD), functional paralysis, and panic, with symptoms that included left-sided “numbing”. Despite treatment with medications, including Risperidone, tics were present every few minutes, reducing school attendance to part-time. Tics improved after nine sessions of I-CBiT over three months, and Risperidone dose was reduced. She returned to school and sports and demonstrated increased independence (using public transport alone). Subsequently, she developed sustained migraines and dissociative episodes, resulting in withdrawal from daily life and bed rest. Organic causes were ruled out. At three month follow-up, there was no presence of tics or dissociative symptoms, with minimal headaches every few weeks. She returned full-time to school/sports and continued to demonstrate independence.

#### 2.2.3. Case 3

A 14-year-old female presented with simple motor and vocal tics, progressing to self-injurious behaviors, hitting, throwing objects, and complex vocalizations. Tic attacks developed within a few months. Tics resulted in reduced school attendance and isolation from peers. She identified as queer and advocated for marginalized groups. She reported being bullied for her sexual orientation and never feeling accepted by her peers, describing herself as an “outsider”. History included social anxiety, panic symptoms, and obsessive-compulsive behaviors (OCBs; hair pulling). She reported ongoing difficulties with initiating and maintaining friendships and was diagnosed with ASD (level 1) during the treatment period. After 10 sessions of I-CBiT over two months, her tics were minimal, with no tic attacks. She returned to school full-time and experienced a reduction in panic attacks from weekly to once every few months, usually during stressful events (social conflict).

#### 2.2.4. Case 4

A 16-year-old female presented with simple motor tics, which progressed to copro-phenomena and self-injurious tics within a few months. Daily functional seizures, functional limb weakness, drop attacks, and paralysis episodes developed a few months later, leading to reduced school attendance and withdrawal from high-level sports. Suicidal ideation and self-harm behaviors were also present. History included GAD, social anxiety, MDD, and a history of trauma with panic and dissociative symptoms. She presented as a high achiever in school/sports. Tics were preceded by multiple international moves for parental employment and the death of a close friend. Her tics showed a marked reduction following 14 sessions of I-CBiT over six months. With 16 further sessions of I-CBiT, other presenting functional symptoms reduced, leading to completion of high school and a move abroad for university.

#### 2.2.5. Case 5

A 14-year-old client who identified as non-binary (biological female) developed a distressing urge to throw a computer. Tic symptoms progressed over the following weeks, resulting in reduced school attendance. History included selective mutism (3–4 years old), GAD, self-harm behaviors, and panic symptoms with worries about social judgment. They presented as a high achiever with perfectionist symptoms. They were a successful online gamer with a large following, noting that tics were an important part of their online identity in that it made them feel accepted and helped manage social anxiety. Following 18 sessions of I-CBiT over eight months, tics reduced in frequency and could be easily suppressed for long periods. They returned to school full-time with no panic symptoms.

#### 2.2.6. Case 6

A 14-year-old female initially developed a shiver accompanied by a right head turn, followed by multiple complex motor tics (throwing pens and stomping) and vocalizations. History included social anxiety, anxiety related to academic ability, a shy and introverted personality type, alexithymia, difficulties with attention and concentration, and low-level disruptive behavior in class. Tics positively affected self-confidence as she felt judgment would be about characteristics beyond her control rather than intrinsic to her personality or identity. She was an avid user of TikTok (engaged in tic-related material) and had a friend with tics. Her tics showed a marked reduction after 11 sessions of I-CBiT over five months. As her tics reduced, she became less engaged with treatment as tics no longer impacted her daily life.

#### 2.2.7. Case 7

A 13-year-old female presented with head nodding and shoulder movements, which increased in frequency (every few seconds) within a few weeks. She presented as a high achiever, demonstrating performance anxiety and alexithymia. History included multiple panic attacks during an assessment/exam week or leading up to one. Tic onset was preceded by a period of significant parental conflict, and she felt a sense of responsibility to dissipate the conflict and care for her siblings. Tic episodes coincided with increased parental attention, resulting in decreased parental conflict. Tics were reduced after 14 sessions of I-CBiT over four months, and she was transitioned into the maintenance phase with diminished tic frequency. Panic attacks also reduced and were mostly limited to exam periods.

#### 2.2.8. Case 8

A 20-year-old female presented with the onset of a forceful head tic (swinging head backward) during a social gathering. Several months later, complex motor/vocal tics and self-injurious behaviors developed. She noticed that some of her tics mimicked those on TikTok (vocalization of “beans”). Due to her tics, she could not cross the road, drive, work, cook, or dress herself. She stopped socializing and spent much time in her bedroom or her long-term girlfriend's home. She presented as a high achiever, demonstrating sporting success. History included social anxiety, GAD, MDD, ASD (level 2), and self-harm behaviors that required hospitalization. Her panic symptoms largely resolved with tic onset, reporting that tics would often interrupt a panic attack. Tics showed a marked reduction after 16 sessions of I-CBiT over seven months. Shortly after, she developed gastro symptoms (unable to eat and vomiting multiple times per day), resulting in several hospital admissions, and was diagnosed as “eating disordered”. The symptoms were treated as functional and resolved with further I-CBiT sessions. She demonstrated progress in all areas of daily living, spontaneously commenced driving, reconnected with peers, and started online dating following the termination of her long-term relationship.

### 2.3. Treatment summary

I-CBiT integrates traditional behavioral tic interventions with third-wave cognitive behavioral therapy to address both the tic symptoms and the underlying stress-arousal system that often triggers the tics [see Table 2 for treatment overview (24)]. According to third-wave models, attempts to modify or control internalized bodily sensations/cognitions perceived as distressing are often ineffective and may exacerbate these experiences. In contrast, accepting or learning to tolerate these internal states can reduce associated distress (25, 26). I-CBiT utilizes traditional exposure and response prevention (ERP), with a focus on urge acceptance. It uses sensory grounding strategies during ERP, assisting with urge tolerance and enhancing attentional switching (21). I-CBiT aims to increase the young person's acceptance of unwanted/distressing interoceptive sensations, here, the urge preceding tics (“tic urge”), while also increasing their control over the behavioral response to the urge. This learning is then generalized to other sensations the young person finds distressing, e.g., anxiety or other somatic symptoms. Improved self-efficacy resulting from effective tic treatment is seen as a key mechanism for change in other areas of functioning, including stress-arousal responses.

**Table 2:** I-CBiT treatment overview.

Phase 1	Phase 2	Phase 3
Psychoeducation	Functional analysis	Cognitive behavioral intervention for arousal and stress
Key components of psychoeducation include: 1) validation of FTLBs as real, distressing, and experienced as uncontrollable. 2) discussion of FTLBs as being an impairment of the functional connections in the neural systems. 3) the role of arousal, stress, and altered brain connectivity within the motor system. 4) formulation of the problem using the client's history and beliefs about the problem; and 5) an outline of the intervention.	Functional analysis includes context, mood, activities, and response from others that occur before, during, and after the FTLBs/exacerbation of FTLBs/other functional symptoms.	Acceptance model is used to address the underlying arousal and stress system, including cognitions, physiology, and behaviors. 1) Emotion: Acceptance strategies applied to “tic urges” are also applied to emotion states (e.g., anxiety, anger, distress) and somatic symptoms that are experienced as unwanted and aversive. This includes awareness of arousal states and willingness to accept fluctuating arousal states without suppression or avoidance. 2) Cognition: Recognition of thoughts as “just thoughts” and use of cognitive defusion strategies. Cognitive challenging is also used, particularly around beliefs about the capacity to tolerate and accept somatic symptoms and urges. 3) Behavior: A hierarchy of goals that the client wants to work towards is created, including activities and events they may have been avoiding due to tics or other symptoms.
Goal setting	Exposure and response prevention with urge acceptance	
1) Short-, medium- and long-term goals are identified, noting losses since the onset of tics and other functional symptoms. 2) Maintaining factors are discussed, including systemic factors.	1) Urge awareness includes mapping urges and sensations that precede tics and other functional symptoms and stress reactions, noting differences and similarities across symptoms. 2) Body scanning to increase awareness of urges, sensations, and somatic symptoms. Imagined or *in vivo* exposure may be used for those unable to recognize preceding urges and sensations. 3) Exposure and response prevention with sensory grounding strategies and attention switching. (i) Clients are encouraged to suppress their motor and vocal tics for increasing duration of time while accepting the urge. (ii) Urges and sensations are likened to a wave that cannot be stopped, but that one can learn to ride. (iii) Clients are given “surfboards” to ride the urge waves. They are encouraged to use these when the urge is intense. (iv) They are encouraged to switch attention back to body sensations and urges once the urge has decreased. (v) Surfboards include: breathing techniques and sensory grounding strategies e.g., 3 things; sounds in speech; sensing feet on the ground. (vi) Clients are encouraged to gradually drop the surfboards and move towards acceptance of the fluctuating urges and sensations. (vii) Clients are encouraged to practice tic surfing with/without the surfboards at home and/or at school at least 10 min×5 days per week.	

Table 2 outlines a summary of the three phases of I-CBiT, which involves core components of psychoeducation, exposure and response prevention with urge acceptance, sensory grounding strategies, and cognitive behavioral intervention targeting the stress-arousal system.

### 2.4. Analysis

To assess the efficacy of I-CBiT in these cases, paired *t*-tests were used to test for significant reductions in total tic severity and impairment (due to tics) post-treatment as measured by the Yale Global Tic Severity Scale [YGTSS (27)].

## 3. Results

All clients engaged well with the I-CBiT treatment. They participated in regular in-session and at-home ERP practice. At-home practice was more regular and effective if the young person had a parent or other adult at home or school to assist them. The intervention was well tolerated, and all clients completed the agreed number of sessions. Therapy endings were all planned, and sessions ended when the tics and co-occurring symptoms were no longer causing significant distress or impacting functioning.

All eight cases showed improvement in three key outcomes post-treatment: (1) tic frequency; (2) tic suppressibility; and (3) YGTSS scores. All cases showed a reduction in tic frequency, with four of the eight cases (1–3, 8) being tic-free post-treatment or only having occasional mild tics once per month. Two cases (4 and 7) had tic-free days or weeks, and the remaining two cases (5 and 6) had tics every couple of hours, with some tic-free days. For those who still experienced occasional tics (*n* = 7), these were mild and simple. Only one case continued to present with complex vocal tics (case 5), albeit with reduced intensity/frequency.

There was marked improvement in tic suppressibility in all cases, with 7/8 cases able to suppress their tics for at least one hour.

**Figure 1:**
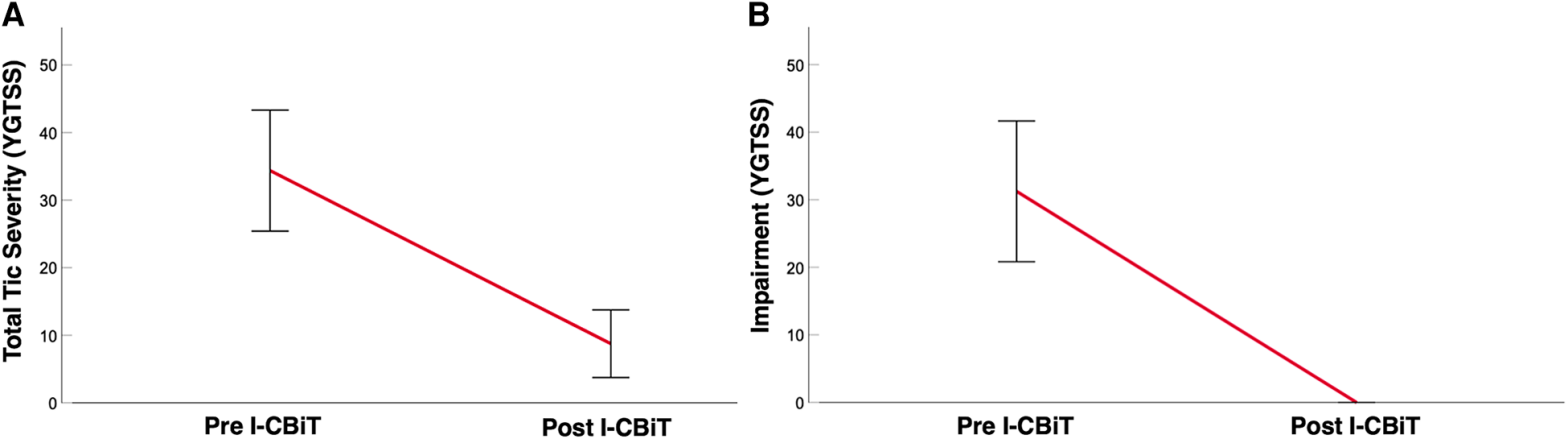
(**A**) Mean total tic severity of the sample as measured by the Yale Global Tic Severity Scale (YGTSS) pre and post I-CBiT intervention. (**B**) Mean impairment (due to tics) of the sample as measured by the YGTSS pre and post I-CBiT intervention.

Paired *t*-tests showed a significant reduction in YGTSS total tic severity scores from baseline, *t*(7) = 5.63, *p* < .001 [*M*_pre_ (*SD*) = 35.00 (10.97); *M*_post_ (*SD*) = 8.50 (6.37)] and a significant reduction in YGTSS impairment scores from baseline, *t*(7) = 7.09, *p* < .001 [*M*_pre_ (*SD*) = 31.25 (12.46); *M*_post_ (*SD*) = 0.00 (0.00)] (see Figures 1A,B).

Improvement in daily living function was evident, with those reporting reduced school attendance (*n* = 4), all returning to school full-time. The panic symptoms present in four cases (3, 5, 7, 8) showed a marked reduction post-treatment. For some cases (1, 2, 4, 8), a drop in mood or manifestation of other functional symptoms was observed alongside the reduction of tics. This presentation supports the role of the stress-arousal system in FTLBs, with tics being a symptom response to stress.

## 4. Discussion

Our findings indicate significant improvement in sudden onset FTLBs and daily functioning following our proposed I-CBiT model. The case examples highlight the stress-arousal system's unique role in presenting FTLBs relative to persistent tic disorders. Central to the present intervention is generalizing tic urge acceptance to the tolerance of other somatic symptoms/sensations. Most of the young people described their ability to accept tic urges as much higher than their acceptance of anxiety or other somatic symptoms. Accordingly, initially treating tics and then generalizing to the underlying stress-arousal states arguably provides a hierarchy of tolerance.

### 4.1. Key trends in clinical presentation

The onset of FTLBs in these cases occurred predominantly in the context of some psychological/physical stressors, with a close temporal relationship. Often, symptoms were accompanied by certain personality or identity characteristics and/or mental health comorbidities. These included ASD symptoms (3, 8), which are typically more prevalent in those with FND (28), social anxiety (2–4, 6, 8), gender incongruence (5), or challenges adapting socially to a new country (1, 4). For 7/8 cases, these stressors led to feelings of social isolation and difference that preceded the tics. Some described long-term bullying and struggling to “belong” amongst their peers, while others noted anxiety relating to social judgment. For most, tics provided unexpected protection against perceived negative judgment and social isolation. Case 6 commented that: “tics allowed me to start conversations. If I was judged, then it was my tics, not me”. 5/8 cases presented with alexithymia or ASD symptoms, suggesting the tics may also “release” otherwise unrecognized and unexpressed stress states. Similarly, case 5 noted that: “tics made me feel accepted and helped manage my social anxiety”, suggesting the critical need for understanding the patient's experience regarding symptoms' function and anxiety's role in maintaining symptom presentations.

### 4.2. Mechanisms for clinical improvement

One strength of our intervention is that it uses the relatively efficacious ERP intervention to increase the client's perception of control over tics (25). We hypothesize that this improves self-efficacy in the client's ability to interrupt/modify their behavior. This learned belief that there can be an alternative response to the experienced urge is then generalized to other somatic symptoms. For example, Case 8 commented when beginning treatment for her functional gastro symptoms: “If I hadn’t done it with tics, no way would I believe it would work, but now I have evidence of it working”.

Second, urge acceptance shares similarities with interoceptive exposure. Accordingly, exposure to the feared internal sensations helps develop beliefs that internal sensations are both safe and tolerable. These new beliefs may be crucial to learning to accept and live well with other uncomfortable arousal states, such as anxiety.

### 4.3. Therapeutic challenges

#### 4.3.1. Maintaining factors

Evidence-based approaches to working with FND include both individual and systemic interventions (17). The reactions/responses to FND symptoms can often be important maintaining factors. For FTLBs, a significant change in the wider system may be the increase in tic-related media on online social media platforms (TikTok) (see 4, 5, 11). The increased prevalence of tics on social media has arguably helped to normalize and destigmatize tics, reducing comorbid mental health difficulties such as anxiety and depression. This increased acceptance also presents additional treatment considerations through unexpected positive reinforcement, such as increased social media following, social acceptance, and the role of tics as a safety behavior for social anxiety. These may, in turn, maintain the tic symptoms and decrease motivation to engage in treatment. Accordingly, realistic and clear goals need to be agreed upon collaboratively early in the therapy.

#### 4.3.2. Symptom replacement

The presentation of new psychopathological symptoms following the remission of FTLBs should be considered. Three of the eight cases (2, 4, 8) presented with new moderate-to-severe functional symptoms as their FTLBs began to resolve, including migraines, severe gastro, or drop attacks. Additionally, case 1 presented with a significant drop in mood and increased suicidality when their tics resolved. Despite this challenge, these new symptoms can be seen as an opportunity to further develop the young person's understanding of their stress-arousal system and symptom management.

### 4.4. Lessons learned

There are several important findings that are worth highlighting. First, while recent criteria for diagnosing FTLBs (12) highlighted the need for distinguishing these symptoms from primary tics, data is still emerging on the nature of the co-occurrence of the two, as was evident in cases 2 and 5. Notably, these cases demonstrated pre-pubertal tics, albeit presenting as mild and simple transitory tics, which were undiagnosed, as opposed to the dramatic onset of severe and sustained “tic-like” movements, with fluctuations linked to stress or anxiety. Second, our clinical experience demonstrates that FTLBs can be successfully treated using an integrated treatment approach that incorporates third-wave cognitive behavioral therapies with a particular focus on teaching urge acceptance and targeting underlying thoughts/emotions that drive anxiety and stress-arousal systems, which are not typically targeted by CBIT. Third, the cases described here highlight the complexity of FND and the need for integrated therapeutic approaches that target both the FTLBs and the underlying maladaptive stress-arousal system. The increased anxiety and suicidality or further functional symptoms observed in some cases following tic resolution necessitated ED presentation/hospital admission. We propose that without appropriate management of the stress-arousal responses as detailed here, alternate functional and psychiatric symptoms may emerge when FTLBs are resolved with consequent cost and burden to the client, their families, and the health care system. Fourth, I-CBiT, as outlined here, may be offered in phases, allowing for flexibility in service delivery. Phases 1 and 2 require a psychologist trained in behavioral interventions for neurodevelopmental tic disorders and experience treating FND alongside more commonly practiced third-wave cognitive behavioral interventions. However, phase 3 can be provided by a generic mental health clinician trained in third-wave cognitive behavioral therapies. Finally, there is an increasing demand for telehealth services for mental healthcare in rural and remote areas. We are currently assessing the acceptability of delivering I-CBiT using an online platform. However, online administration presents unique challenges. For example, it is often more effective and safer to be in the same room as the client when conducting ERP, particularly when the client is experiencing intense tics or other functional episodes. Using an online administration can also make it more challenging to engage and work with the system around the client, particularly the family system.

### 4.5. Limitations

Some limitations should be acknowledged. First, all the cases reported here were adolescent/young adult biological females. While this case series sample is reflective of the sex composition of the patients who presented to our clinical practices for treatment and is largely consistent with the FTLB presentations in the population, with an estimated female:male ratio of 9:1 (20, 29, 30), future investigations assessing the efficacy of I-CBiT in male patients, different age groups, and geographic locations would be valuable. Second, while standardized measures of tic severity, suppressibility, and impairment were assessed, given the retrospective nature of the data ascertained for this case series from a tic-specific clinic, only standardized measures of tic severity and impairment were accessible and precluded the availability of standardized measures of stress and anxiety. Future examination of this model in a larger sample with standardized assessments of stress, anxiety, and somatic symptoms pre- and post-treatment would further inform their role in FTLB presentation. Finally, as previously mentioned, given the observational nature of this work, the applicability of I-CBiT to additional FTLB presentations is not yet fully understood. However, at present, there are no apparent clinical features that would diminish the generalizability of these findings.

### 4.6. Conclusion

We highlighted the unique features of FTLBs and the role of biological or environmental stressors as triggers in predisposed individuals. Our proposed therapeutic model, I-CBiT, focuses not only on traditional behavioral techniques but also on cognitive elements of symptom presentation, including acceptance and coping. It illustrates the value of generalizing tic urge acceptance to the acceptance of other underlying stress-arousal symptoms. Rather than treating tics in isolation, we suggest increasing self-efficacy about one's ability to interrupt behaviors and motor responses is critical. This, coupled with modifying negative beliefs about unwanted interoceptive sensations, can potentially bring about more generalized and long-term change. Accordingly, these cases highlight the need for more integrated care practices and greater interaction between multidisciplinary services for comprehensive management covering all areas of FND.

## Data Availability

The datasets presented in this article are not readily available because due to client confidentiality, raw clinical data cannot be made publicly available. Questionnaires used by the clinical team are available on request. Requests to access the datasets should be directed to amanda.maxwell@unsw.edu.au.
